# Association between clinical frailty, illness severity and post-discharge survival: a prospective cohort study of older medical inpatients in Norway

**DOI:** 10.1007/s41999-021-00555-8

**Published:** 2021-08-21

**Authors:** Andreas Engvig, Torgeir Bruun Wyller, Eva Skovlund, Marc Vali Ahmed, Trygve Sundby Hall, Kenneth Rockwood, Anne Mette Njaastad, Bjørn Erik Neerland

**Affiliations:** 1 Department of Internal Medicine, Oslo University Hospital (OUS), Oslo, Norway; 2 Department of Nephrology, Division of Medicine, Oslo University Hospital, Ullevål Hospital, Nydalen, PO Box 4956, 0424 Oslo, Norway; 3 Department of Geriatric Medicine, OUS, Oslo, Norway; 4 Institute of Clinical Medicine, University of Oslo (UiO), Oslo, Norway; 5 Department of Public Health and Nursing, Norwegian University of Science and Technology, NTNU, Trondheim, Norway; 6 Geriatric Medicine, Dalhousie University, Halifax, NS Canada

**Keywords:** Clinical Frailty Scale, Frailty index, Survival analysis, New Early Warning Score, Acute care, Hospital complications

## Abstract

**Purpose:**

Study associations between frailty, illness severity and post-discharge survival in older adults admitted to medical wards with acute clinical conditions.

**Methods:**

Prospective cohort study of 195 individuals (mean age 86; 63% females) admitted to two medical wards with acute illness, followed up for all-cause mortality for 20 months after discharge. Ward physicians screened for frailty and quantified its degree from one to eight using Clinical Frailty Scale (CFS), while clinical illness severity was estimated by New Early Warning Score 2 (NEWS2) and laboratory illness severity was calculated by a frailty index (FI-lab) using routine blood tests.

**Results:**

CFS, NEWS2 and FI-lab scores were independently associated with post-discharge survival in an adjusted Cox proportional hazards model with age, ward category (acute geriatric and general medical) and comorbidity as covariates. Adjusted hazard ratios and 95% confidence intervals were 1.54 (1.24–1.91) for CFS, 1.12 (1.03–1.23) for NEWS2, and 1.02 (1.00–1.05) for FI-lab. A frailty × illness severity category interaction effect (*p* = 0.003), suggested that the impact of frailty on survival was greater in those experiencing higher levels of illness severity. Among patients with at least moderate frailty (CFS six to eight) and high illness severity according to both NEWS2 and FI-lab, two (13%) were alive at follow-up.

**Conclusion:**

Frailty screening aided prognostication of survival following discharge in older acutely ill persons admitted to medical wards. The prognostic value of frailty increased when combined with readily available illness severity markers acquired during admission.

**Supplementary Information:**

The online version contains supplementary material available at 10.1007/s41999-021-00555-8.

## Key summary points

**Aim:**

To assess impact of frailty screening and two markers of illness severity on survival following discharge from the hospital.

**Findings:**

Independently of age, ward (acute geriatric and general medical) and comorbidity, both higher degree of frailty and illness severity associated with reduced survival probability following discharge. The impact of frailty on survival was higher in those experiencing high clinical and laboratory illness severity.

**Message:**

The prognostic value of frailty screening increased when performed in conjunction with two markers of illness severity.

**Supplementary Information:**

The online version contains supplementary material available at 10.1007/s41999-021-00555-8.

## Introduction

Older adults admitted with acute clinical conditions have an increased, but variable mortality risk following hospitalization. Assessment of frailty, an age-associated state characterized by increased vulnerability to stressors, may help risk stratification [1, 2]. In a 2021 study from Brazil, the frailty screening instrument, Clinical Frailty Scale (CFS) was shown to predict prognosis together with measures of clinical illness in patients hospitalized with COVID-19 [3].

CFS quantifies the degree of frailty by assessing the inpatient’s functional status at least two weeks prior to admission. Research indicates that sound clinical judgement and history taking are needed for frailty screening by CFS to be informative for prognosis: In a United Kingdom (UK) study, Shrier and colleagues found that rapid frailty screening at emergency department triage had low predictive value in acutely ill persons ≥ 65 years later admitted to medical wards [4]. When scored by a ward physician following admission to the ward, however, CFS correlated strongly with length of stay and inpatient mortality. The results suggested that ward clerking is a suitable setting to perform frailty screening.

The prognostic value of frailty by CFS-scoring depends not only on the quality of its assessment, but also by its relationship with illness severity. In 2016, Romero-Ortuno and colleagues showed in retrospective analysis that CFS scores as well as illness severity measured by an early warning score were associated with survival. The impact of illness severity was particularly high among those living with severe frailty. In addition to routinely collected clinical data informing clinical illness severity such as early warning scores, derangements in blood tests collected upon hospital admission may yield prognostic information. Ellis and colleagues [5] quantified laboratory illness severity by a frail index (FI-lab) approach using routine blood test results from 2254 hospital admissions, and showed that FI-lab as well as CFS scores associated with mortality. To this date, no study has prospectively evaluated the impact of clinical and laboratory illness severity together with degree of frailty on longer-term mortality risk.

Here, we instructed ward physicians to assess degree of frailty using CFS in a sample of older acute inpatients recruited from two medical wards (acute geriatric and general medical) in Norway. Our objective was to assess the prognostic value of CFS, as well as New Early Warning Score 2 (NEWS2)- and FI-lab-scores obtained as part of daily practice in terms of survival following discharge. We hypothesized that higher CFS and illness severity scores were associated with reduced 20-month survival probability following discharge, and further assessed interactions between the frailty and illness severity scores on survival.

## Materials and methods

### Study design and eligibility criteria

**Fig. 1:**
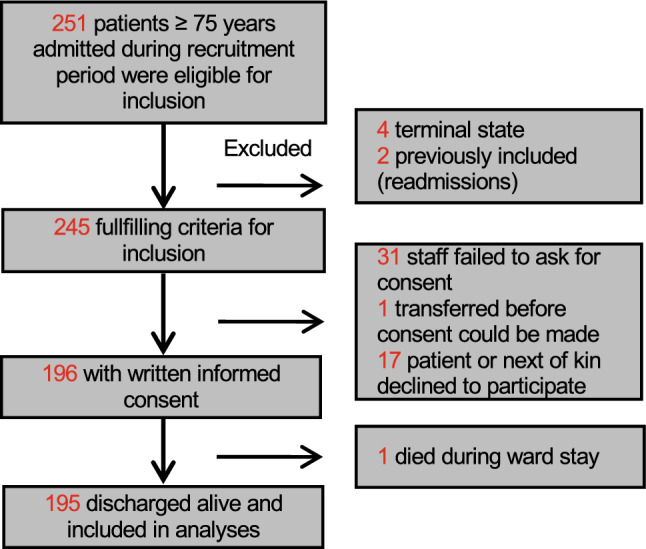
Flowchart of patient recruitment

We conducted a prospective observational study with a recruitment period from March to May 2019. Patients aged ≥ 75 years admitted acutely to two medical wards (acute geriatric and general medical) at Oslo University Hospital during the recruitment period were eligible (Fig. 1). The catchment area for Oslo university hospital covers six districts in Oslo amounting to a population of about 300.000. Older people admitted acutely to the two wards present with a diverse range of medical conditions and frailty. We excluded previously enrolled patients who were readmitted during the study period and those who were terminally ill (i.e., a CFS score of nine).

### Data collection

Baseline data were obtained from the medical records. We registered disease burden by calculating the Charlson Comorbidity Index (CCI) [6]. The CCI is based on discharge diagnoses, as coded by the 10th version of the WHO International Classification of Diseases (ICD-10). Hospital delirium, either present at the time of admission or developing after admission, was identified by an experienced delirium researcher (BEN) through a validated and reliable medical record review approach, based on trigger words and phrases [7].

### Frailty assessment

To classify the degree of frailty, we used the Norwegian CFS translation by Hans Flaatten and Brit Sjøbø from 2018. CFS is a frailty screening tool which consists of nine images with accompanying brief clinical descriptors, categorizing individuals from robust (CFS 1) to very severely frail (CFS 8) or terminally ill (CFS 9) [8]. The Norwegian translation and the images used for each category are shown in Online Resource 1. In the most recent version, a person with a CFS score of four is considered living with (very mild) frailty, whereas the older version used herein requires a CFS score of five to be considered living with (mild) frailty. We used both cut-offs to estimate frailty prevalence. As the purpose of this study was to evaluate the impact of frailty, we excluded patients with CFS category nine. Attending ward physicians (general internal medicine as well as geriatric medicine) screened and quantified frailty using the CFS. The physicians did not have previous experience with the CFS, but received a 30-min training session in its use prior to the recruitment period. They were told to perform the CFS as part of the initial clerking of the patient following ward admission (typically within the first 72 h of the hospital stay), and based on their judgement of the patient´s functional status before admission using clinical information at hand.

Independently of the CFS scores, a comprehensive clinical deficit-accumulation frailty index (range from zero to one; higher values indicate greater degree of frailty) based on that of Kim et al. [9], was calculated retrospectively by a researcher (BEN) using available data retrieved from medical record review.

### Illness severity

Clinical illness severity on admission and during the hospital stay was estimated by the standardized scoring system NEWS2 [10]. The score can range from zero to 20 (higher = worse severity), and is derived from assessment of vital signs. We selected the maximum recorded NEWS2 score during the stay for the analyses, and used a conventional cut-off of ≥ 5 to indicate high clinical illness severity.

Blood test results reflecting illness severity were estimated by means of a laboratory frailty index (FI-lab) [11]. The present FI-lab variable was calculated from 14 blood tests routinely performed on any acute medical inpatient admitted to our hospital, using the hospital’s laboratory reference values as cut-offs (Online Resource 2). We used blood test results from the time of hospital admission, as the subsequent number of blood tests varies substantially among patients after the initial admission blood draw. A cut-off score of ≥ 0.45, commonly employed to characterize “severe” cases on a frailty index continuum [12, 13], was used as a marker of high laboratory illness severity. We also extracted serum albumin, CRP and estimated CRP/albumin values for each patient to compare their relationship with survival with that of FI-lab.

### Primary outcome

Our primary outcome was time from discharge to death of any cause. We followed patients until the end of the following year (December 23, 2020). Patients still alive at the end of follow-up were censored.

### Ethics

The study was conducted in accordance with the Declaration of Helsinki and approved by the Regional Committee for Ethics in Medical Research in Norway (REK 2018/2554-7). Written, informed consent was obtained for all participants or, if participants did not have capacity to consent, from substitute decision-makers.

### Statistical analyses

We calculated Pearson’s correlation coefficients to assess bivariate relationships between CFS scores, the comprehensive clinical frailty index computed by the researcher, as well as the NEWS2 and FI-lab scores. We tested for multicollinearity between CFS, NEWS2 and FI-lab by means of variance inflation factors. We also tested associations between the degree of frailty and selected admission-related variables, including number of drugs, length of stay (LOS), in-hospital delirium, and discharge destination. Here, normality assumptions were confirmed by visual inspection of histograms and residual plots. Deviation from normality was only seen for hospital LOS. We performed Chi-square tests for trend for the categorical outcomes, and linear regressions for ordinal and continuous outcomes, except for LOS. We tested the association between degree of frailty and LOS using a non-parametric Jonckheere-Terpstra test of trend.

To test if degree of frailty and the two indices of illness severity were independently associated with survival probability following discharge, we first fitted Cox proportional hazards models using the full scale CFS variable (ranging from one to eight), NEWS2 and FI-lab scores, as well as pre-defined covariates (age, ward type and CCI). Log minus log survival curves for CFS, NEWS2 and FI-lab tertiles were mostly parallel, suggesting proportionality of hazards. Hazard ratios (HR) were estimated for each of the covariates in separate univariate Cox proportional hazards models, as well as in an adjusted model including all six variables.

Survival was then compared between groups of CFS, as well as NEWS2 and FI-lab categories by the Kaplan–Meier estimator. For the main analyses, the following four categories of CFS scores were used: CFS one to three (fit to well, i.e., living without frailty), CFS four to five (living with very mild to mild frailty), CFS six (living with moderate frailty) and CFS seven to eight (living with severe to very severe frailty). For NEWS2 and FI-lab, patients were categorized into four groups according to their either high or low severity score on the two measures.

Next, we tested the interaction between degree of frailty and combined illness severity by fitting a Cox proportional hazards model using CFS scores and the categorical variable including the four groups of illness severity (high or low) as covariates.

To compare our FI-lab variable with two established laboratory illness severity markers [14], albumin and CRP/albumin-ratio, we fitted the same Cox proportional hazards models, but used either albumin or CRP/albumin-ratio as covariates instead of FI-lab. We also compared survival according to FI-lab, CRP/albumin-ratio, and albumin tertiles by the Kaplan–Meier estimator.

Statistical analyses were performed using SPSS Statistics version 27 (IBM Corp., Armonk NY). The level of statistical significance was set to *p* < 0.05.

## Results

### Baseline characteristics

A total of 251 patients were admitted during the recruitment period. A total of 195 patients were included, representing 80% of those matching inclusion criteria (Fig. 1). There were 122 (63%) females, and the mean age was 86 (standard deviation (SD) 5.7, range 75–100) years (Table 1). Patients admitted to acute geriatric medicine were older and living with higher degree of frailty, compared with those admitted to general medicine (independent samples *t*-tests, *t* > 2.6, *p* < 0.02). Overall, the percentage of patients classified as living with frailty was 88% and 67% when applying CFS frailty-thresholds of ≥ four and ≥ five, respectively. CFS scores obtained by ward physicians correlated strongly with a comprehensive clinical frailty index calculated retrospectively by the researcher (Pearson’s *r* = 0.73, *p*-value < 0.001). 71% were classified as at least mildly frail using a commonly used frailty index threshold of 0.2. A frailty index threshold of > 0.3, indicated that 55% were living with at least moderate frailty, whereas a CFS threshold of > five indicated 43%. Online Resource 3 shows patient characteristics in relationship to different degrees of frailty.

**Table 1:** Baseline characteristics of total study sample (*N* = 195)

	Mean (SD)	*N* (%)	Median	IQR	Min	Max
Mean age, years	86.3 (5.7)		8	8	75	100
Female, *N* (%)		122 (63)				
Charlson Comorbidity Index	1.9 (1.7)		2	2	0	9
Length of hospital stay, days	8.5 (8.6)		7	5	0	88
Frailty status						
CFS score	5.2 (1.5)		5	2	1	8
CFS 1–3 (fit and well)		23 (12)				
CFS 4 (very mild frailty)		42 (22)				
CFS 5 (mild frailty)		46 (24)				
CFS 6 (moderate frailty)		44 (23)				
CFS 7–8 (Severe/very severe frailty)		40 (21)				
Illness severity						
NEWS2 score	4.9 (2.7)		4	4	0	13
High clinical illness severity*		95 (49)				
FI-lab score	0.37 (0.15)		0.36	0.21	0.0	0.71
High laboratory illness severity**		53 (27)				
Both high clinical and laboratory illness severity		26 (13)				

*CFS* Clinical Frailty Scale; *CI *confidence interval; *FI-lab* Frailty index of 14 routine blood tests; *IQR* interquartile range; *N*  number of participants; *NEWS2 *New Early Warning Score 2, maximum value during hospital stay; *SD* Standard deviation

*High clinical illness severity = NEWS2-score ≥ 5

**High laboratory illness severity = FI-lab value ≥ 0.45

A total of 95 patients (49%) exhibited high clinical illness severity during the hospital stay, indicated by a maximum NEWS2-score ≥ 5, whereas 53 (27%) had high laboratory illness severity, indicated by a FI-lab score ≥ 0.45. FI-lab did not correlate significantly with NEWS2 (*r* = 0.098, *p* = 0.17). CFS correlated moderately with NEWS2 (*r* = 0.28, *p* < 0.001), suggesting increased clinical illness severity in patients living with higher degree of frailty. CFS showed weak non-significant correlation with FI-lab (*r* = 0.13, *p* = 0.062). Low variance inflation factor values (1.01, 1.02, and 1.08, for CFS, NEWS2 and FI-lab as independent variables, respectively) suggested minimal multicollinearity.

### Primary outcome: CFS and both indices of illness severity are associated with all-cause post-discharge mortality independent of age, ward type and comorbidity

**Fig. 2:**
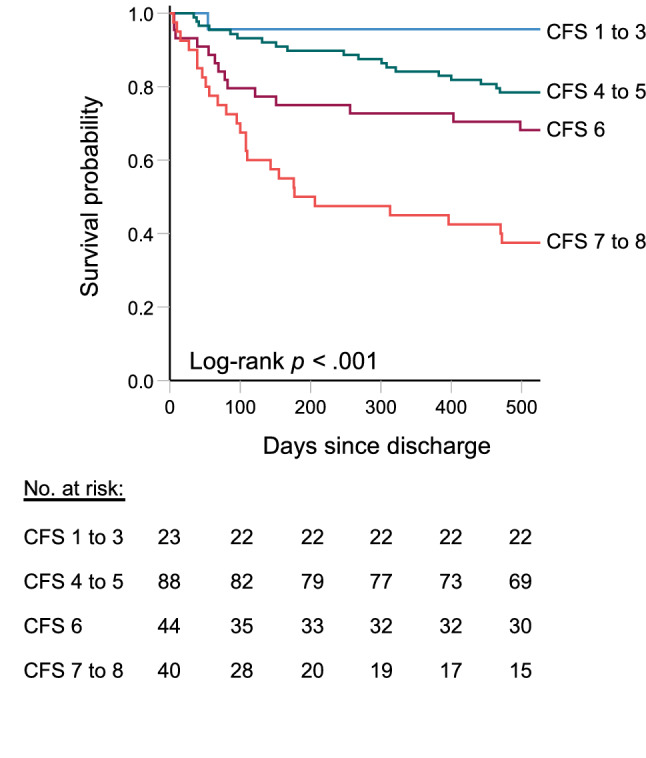
Kaplan–Meier curves illustrating estimated survival probabilities within follow-up according to different degrees of frailty. The survival curves according to CFS categories indicating no (1–3), very mild to mild (4–5), moderate (6) or severe to very severe (7–8) frailty were statistically different (Log-rank test, *p* < 0.001)

**Fig. 3:**
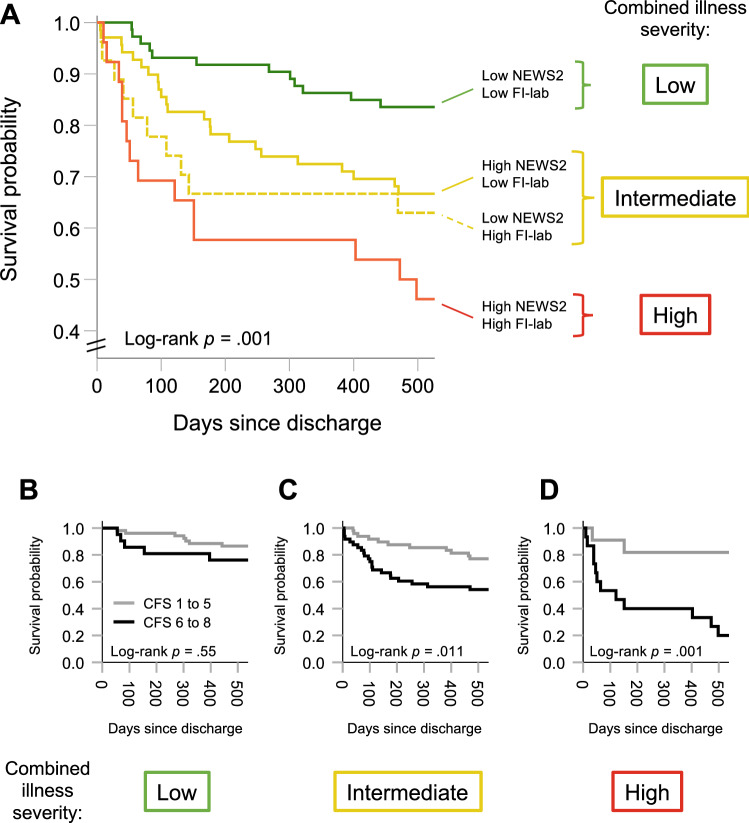
**a** Kaplan–Meier curves display estimated survival probabilities within follow-up according to four categories of NEWS2 and FI-lab scores (high or low). Visual inspection suggests more favourable survival for patients with less severe illness. A log-rank test confirmed a significant difference between the survival curves. To visualize the interaction between NEWS2 and FI-lab categories and frailty, subjects were divided based a combined illness severity pattern emphasized by color: we considered low NEWS2 and FI-lab scores (green curve) as low; high NEWS2 (straight line) or FI-lab (dotted line) as intermediate (yellow curves); and high NEWS2 and FI-lab as high combined illness severity (red curve), respectively. **b**–**d** shows Kaplan–Meier plots of estimated survival probabilities for subjects living with no to mild (CFS 1–5; grey curves) or moderate to severe (CFS 6–8; black curves) frailty––stratified according to low, intermediate or high combined illness severity. Visual inspection of the curves indicates negligible differences in survival across categories of illness severity among patients living with no or mild frailty. The curves further suggests that the impact of living with moderate to severe frailty on survival probability is most pronounced in the face of more severe illness

Among the 195 patients discharged from the hospital and included in the analyses, there were 49 deaths within 1 year (25%) and 63 deaths within the end of follow-up (32%). Median follow-up time from discharge to death or censoring was 588 days (Interquartile range (IQR) 321 to 618). Table 2 shows results from the Cox proportional hazards models where CFS, NEWS2 and FI-lab scores were independently associated with survival probability, when adjusting for age, ward (acute geriatric or general medical) and comorbidity. In Fig. 2, the Kaplan–Meier survival curves illustrates how survival probability decreases with higher degrees of frailty according to four categories of CFS. Full-scale CFS survival curves are shown in Online Resource 4. Figure 3a shows Kaplan–Meier survival curves according to degree of combined illness severity as represented by the four possible combinations of high or low NEWS2 and FI-lab scores.

**Table 2:** Associations estimated by Cox proportional hazards models with death within follow-up for the full sample (*N* = 195)

Variable	Mortality	
	Hazard ratio (95% CI)	
	Unadjusted model	Adjusted model
Age	1.08 (1.03–1.12)	1.06 (1.02–1.11)
Ward	1.09 (0.67–1.79)	1.70 (0.97–3.00)
CCI	1.47 (1.31–1.64)	1.33 (1.17–1.52)
CFS	1.70 (1.39–2.08)	1.54 (1.24–1.91)
NEWS2	1.14 (1.04–1.24)	1.12 (1.03–1.23)
FI-lab*100	1.04 (1.02–1.05)	1.03 (1.00–1.05)

The adjusted model included all six variables listed in the first column as covariates. Ward placement (acute geriatric medicine or internal medicine) was coded as a categorical variable with acute geriatric medicine as reference

*CCI* Charlson Comorbidity Index; *CFS* Clinical Frailty Scale; *CI* confidence interval; FI-lab*100 Frailty index comprised of 14 routine blood tests multiplied by a factor of 100; *NEWS2* New Early Warning Score 2, maximum value during hospital stay

We also included albumin and CRP/albumin-ratio instead of FI-lab as markers of laboratory illness severity in separate Cox proportional hazards models; these co-variates failed to reach statistical significance in the adjusted analyses (*p* values > 0.05). Differences between FI-lab, albumin, and CRP/albumin-ratio tertiles and survival probability are illustrated by means of separate Kaplan–Meier survival curves in Online Resource 5.

### With advancing degrees of frailty, survival probability is reduced as illness severity increases

In a Cox proportional hazards model including CFS scores as a scale variable and combined illness severity classified according to high or low NEWS2 and FI-lab scores (see Fig. 3a), we found a statistically significant frailty × illness severity interaction (*p* = 0.003). The interaction result suggests that the impact of frailty on survival is greater in those experiencing higher levels of illness severity. To visualize the pattern of results, we further classified patients according to three categories of combined illness severity guided by the survival curves in Fig. 3a: “low”, “intermediate” and “high”. Using the three categories as strata, we plotted Kaplan–Meier survival curves for frailty status in Fig. 3b–d. Here, frailty was dichotomized according to recent convention (CFS one to five versus six to eight) due to the low number of events. Among persons living with at least moderate frailty (CFS score greater than five), 100-day survival probabilities according to low, intermediate and high combined illness severity were 86%, 77% and 53%, respectively. Among patients with CFS scores greater than five, two individuals out of 15 (13%) with high combined illness severity during hospital stay were alive at the end of follow-up.

## Discussion

We explored the impact of frailty screening and two illness severity markers and their interactions on survival following discharge in a prospective cohort of acutely ill medical inpatients aged 75 and older. There was a strong association between degree of frailty, as evaluated by ward physicians during initial clerking of the patient, and survival. Survival probability decreased by 44% for each increment step on the CFS in adjusted analysis. Survival was further associated with the patients’ illness severity—both assessed clinically using NEWS2 as well as by indexing derangements in 14 routinely analyzed blood tests (e.g., hemoglobin, creatinine, etcetera). Whereas clinical indices of illness severity such as early warning scores likely reflects the physiological response to acute illness, biochemical derangements might encompass acute and more chronic illness-related processes. The combined use of early warning scores and grading of biochemical derangements (FI-lab) to evaluate illness severity and a frailty screen measure in assessment of longer-term prognosis is novel. Our results suggest that the combined assessment is particularly informative, and improves prognostic yield in older medical inpatients compared with either measure alone. Individuals living with severe–very severe frailty who experience severe illness—as assessed by both NEWS2 and FI-lab––had a poor prognosis (about 50% 100-day survival probability).

We found that survival probability in older individuals who are acutely ill decreases with increasing levels of frailty as measured by CFS performed by ward physicians. The finding is in accordance with a growing body of literature, accumulating before and following COVID-19 [2, 4, 5, 15–17]. Compared with other studies of individuals admitted to medicine before the COVID-19 pandemic, the mortality rates in the present study were relatively low; the 30-day and six-month mortality rates were 4% and 20%, respectively. In a study of older hospitalized adults referred to medicine by Pulok and colleagues, the comparable rates were 17% and 34%, respectively. The discrepancy in death rate might in part be explained by the higher number of relatively fit patients in our study (33% had a CFS score of 1 to 4 compared with 24% in [17]) and the fact that we excluded patients with terminal illness. Further, we assessed relatively stable inpatients in medical wards who survived the hospital stay. In a retrospective analysis of older hospitalized adults with CFS scores from one to eight from Cambridge, UK, Romero-Ortuno reported a 30-day mortality proportion of 6.7%, with significant differences in survival according to degree of frailty. The present study was underpowered to evaluate 30-day mortality according to frailty category, but suggests that CFS––as well as illness severity––are also related to longer-term prognosis.

Although Modified Early Warning Score (MEWS) and NEWS2 are the most widely studied instruments to evaluate illness severity during hospital admissions, the optimal scoring method is unknown [18]. Using MEWS- and CFS scores obtained in the Emergency department, Romano-Ortuno et al., showed that both measures corresponded to 30-day survival among older inpatients in retrospective analysis––prompting a call for a prospective study. In Norway, the hospital use of NEWS2 is mandated nationwide and automatically derived by widespread implementation of electronic medical record systems. In prediction of mortality, the score including the most deranged values is likely the most informative [18]. Here, we show that for every 1-point increment in the worst NEWS2-score during hospital stay, the mortality hazard ratios during a 20-month follow-up period increased by 14% (unadjusted HR 1.14 (1.04–1.24). NEWS2 correlated moderately with CFS scores, suggesting that patients with higher levels of frailty were sicker––a finding in agreement with others [2, 19]. Although multicollinearity assessment between NEWS2 and CFS was negative, it should be noted that the HR estimate for NEWS2 approached 1.00 in adjusted analysis (1.09 (1.00–1.20)). As noted by Romano-Ortuno [2], the correlation between illness severity and frailty may represent lead-time bias––where patients living with frailty present later to the hospital, as well as a poor ability of the frail to compensate for acute illness. Our finding suggests that an observation of extremes in both NEWS2 and CFS warrants high levels of attention to the patient’s care plan and its intensity.

For the present study, we hypothesized that additional, routinely collected patient data, besides early warning scores, are related to prognosis. One way of operationalizing illness severity based on blood tests is the FI-lab, developed by Howlett and coworkers in a community setting [11] using the accumulating deficits approach [20]. In 2020, Ellis showed that FI-lab may be informative when applied to inpatients: Their FI-lab variable comprised routinely collected blood tests and predicted risk of many adverse hospital outcomes, including death. We calculated a FI-lab using blood tests performed on any admitted patient to our medical wards. The resulting variable did not correlate with NEWS2, and was independently associated with survival. In the adjusted model, for each increment (e.g., from 0.25 to 0.26) in the FI-lab, the mortality hazard ratios increased by 3% (95% confidence interval 1.01–1.05). Inpatient FI-lab scores may be calculated through electronic health systems, making FI-lab a promising variable to test in larger-scale studies; this is further corroborated by FI-lab’s strong relationship with survival compared with traditional laboratory biomarkers of survival, e.g., CRP/albumin-ratio and albumin (Online Resource 5). Our results showed that the combination of high NEWS2- and FI-lab-scores is associated with worse prognosis, compared with either alone––and in particular for those living with moderate to severe frailty. Further studies are needed to validate these results and see if having both CFS, NEWS2 and FI-lab results at hand is indeed useful to identify those a highest risk, and if it can be used to guide clinical practice.

Strengths of the present study included the prospective design and real-life setting suggesting that frailty screening is useful when performed by ward physicians. There were few missing data, and an 80% inclusion rate points to a relatively representative sample. 16 patients had up to six missing blood tests, which might have impacted the value of the FI-lab-variable. Leaving these subjects out, running the analyses for the 180 subjects with complete blood test results only did, however, not change the results. A limitation is the use of only 14 blood test results to construct the FI-lab variable. More precise estimates of individual laboratory illness severity might have been obtained using a wider range of blood test results. The age and gender-adjusted association between FI-lab and death during follow-up was, however, comparable between our study (HR 1.04, 95% CI 1.02–1.05) and that of the FI-lab validation study by Howlett (HR 1.03, 95% CI 1.02–1.04) [11]. CFS’s ability to measure frailty depends on proper test administration and validated translation schemes when a foreign language is used [21, 22]. Although correctly assessing frailty during the acute phase might be challenging, we sought to improve test administration and classification accuracy by training CFS raters at the beginning of the study, putting a particular emphasis on evaluating the baseline state prior to admission [21]. We also believe, based on previous research [4], that assigning CFS assessment to clinically relatively experienced ward physicians during the initial clerking of the patient as compared to time-constrained healthcare workers in the emergency department may further have improved classification accuracy, although this was not formally tested in our study. It should be noted that the CFS translation used herein lacks a formal validation study. The Norwegian CFS translation was, however, performed according to the validated method described by French CFS translators [22] and has shown to have good inter-rater reliability when used in ICU patients [23]. Further, the present results show that data obtained by the Norwegian CFS version associate with a range of clinical endpoints and strongly correlate with a validated frailty index, giving support to its continued use in Norway. Finally, as the study was conducted before the outbreak of the coronavirus disease pandemic, the results may not be applicable for patients with COVID-19.

## Conclusion

Two readily available illness severity measures accessible by electronic medical records in combination with frailty screening by ward physicians relate to prognosis following discharge among our patients. The data implicate that combining information about a patient’s baseline state using CFS with severity of present illness may be particular useful when planning for discharge. E.g., in a very frail patient facing severe illness, addressing their advanced care plans and ceilings of care may be important. Our data further suggest that those who are living with frailty, but experience less severe illness have relatively good long-term prognoses. Here, CFS might help improve the discharge plan by better expressing the need for home assistance or rehabilitation corresponding to the patient’s current degree of frailty. Overall, given its ability to help stratify risk of adverse health outcomes within a reasonable time frame, we believe frailty screening has potential for implementation in our everyday practice. Nationwide health services, such as that of the UK has already implemented frailty assessment at the front door [24]. Our results imply that a larger scale evaluation of its potential for implementation in Norway is warranted.

## Supplementary Information

Below is the link to the electronic supplementary material.

Supplementary file1 (PDF 1095 KB)

Supplementary file2 (PDF 203 KB)

Supplementary file3 (PDF 227 KB)

Supplementary file4 (PDF 88 KB)

Supplementary file5 (PDF 152 KB)

## Data Availability

Data may be available upon reasonable request to the authors. Availability is dependent on approval from the Regional Ethics Committee for medical research in the South-East of Norway and the data protection officer at Oslo University Hospital.

## Code availability

SPSS syntax for computation of frailty index (FI)-lab variable and statistical analyses will be provided upon reasonable request.
