# Supplementary material for: Association between clinical frailty, illness severity and post-discharge survival: a prospective cohort study of older medical inpatients in Norway
Source: Eur Geriatr Med. 2021 Aug 21;13(2):453–61. doi: 10.1007/s41999-021-00555-8 (PMC8379589; doi:10.1007/s41999-021-00555-8)
Supplement: Supplementary file 1 — Supplementary file1 (PDF 1095 KB) [file 41999_2021_555_MOESM1_ESM.pdf]

Online Resource 1: Norwegian CFS translation used for the present study

## Clinical Frailty Scale Norsk versjon

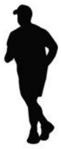

**1. Veldig sprek**. Personer som er robuste, aktive, energiske og motiverte. De trener vanligvis regelmessig, og er blant de mest spreke i aldergruppen.

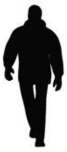

**2. Sprek**. Personer som ikke har aktive sykdomssymptom, men er mindre spreke en kategori 1. De kan trene og være veldig aktive av og til, eks sesongpreget.

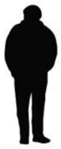

**3. Klarer seg bra**. Personer hvis medisinske problem er godt kontrollert, men er ikke regelmessig aktive foruten vanlig gange.

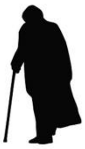

**4. Sårbar**. Selv om de ikke er avhengig av daglig hjelp fra andre, vil symptomer begrense aktiviteter. De klager ofte over at de «svekkes» og blir fort slitne og trøtte ila dagen.

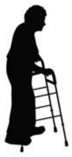

**5. Lett skrøpelig**. Disse er klart langsomme, og trenger hjelp til mer komplekse daglige aktiviteter (finanser, transport, vask og rengjøring, medikamenter). Vanligvis vil gruppen i økende grad ha problem med å handle eller gå utenfor huset alene, lage måltider og ordne i hjemmet.

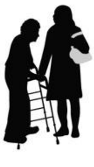

**6. Moderat skrøpelig**. Personer som må ha hjelp til alle aktiviteter utenfor hjemmet og med å stelle hjemmet. De har problem med trapper, trenger hjelp til bading og kan trenge litt hjelp til å kle seg.

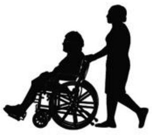

**7. Alvorlig skrøpelig**. Disse personer er helt avhengig av hjelp til personlig pleie både av fysiske eller kognitive årsaker. De er ellers stabile, og har ikke stor risiko for å dø ila en 6 måneders periode

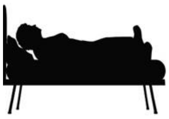

**8. Svært alvorlig skrøpelig**. Held avhengig til alle gjøremål, nærmer seg livets slutt. Typisk er at de ikke kommer seg selv etter mindre akutte hendelser.

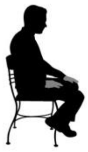

**9. Terminalt syk**. I livets sluttfase. Kategorien kan brukes der leveutsiktene med stor grad av sikkerhet er < 6 mnd. Selv om de ikke kan kalles «frail» (skrøpelig)

**Vurdering av skrøpelighet hos mennesker med demens.**

Grad av skrøpelighet sammenfaller med grad av demens. Vanlige **symptom på mild demens** omfatter det å glemme detaljer rundt en nylig hendelse, men fremdeles evne å kunne huske selve hendelsen, å gjenta de samme spørsmålene / hendelsene og sosial tilbaketrekking.

I **moderat demens** er korttidsminnet svært svekket, selv om de tilsynelatende kan huske hendelser fra tidligere i livet. De kan ivareta egenomsorgen under veiledning.

Ved **alvorlig demens** må de ha hjelp til all egenomsorg.

K. Rockwood et al. A global clinical measure of fitness and frailty in elderly people. CMAJ 2005;173:489-495.

Oversatt til norsk januar 2018 av Hans Flaatten (dr.med) og Britt Sjøbø (MSc), Intensivmedisinsk seksjon, Kirurgisk serviceklinikk, Haukeland Universitetssjukehus, Bergen, Norge.

Supplementary material for «Association between clinical frailty, illness severity and post-discharge survival: A prospective cohort study of older medical inpatients in Norway», published in European Geriatric Medicine.
Authors: Andreas Engvig, Torgeir Bruun Wyller, Eva Skovlund, Marc Vali Ahmed, Trygve Sundby Hall, Kenneth Rockwood, Anne Mette Njaastad, and Bjørn Erik Neerland.
Corresponding Author: Andreas Engvig, M.D., Ph.D., andreas.engvig@gmail.com

**©2009. Version 1.2_EN. All rights reserved. Geriatric Medicine Research, Dalhousie University, Halifax, Canada. Permission granted to copy for research and educational purposes only. Translated with permission to Norwegian by Hans Flaatten and Britt Sjøbø, Intensivmedisinsk seksjon, Kirurgisk serviceklinikk, Haukeland Universitetssjukehus, Bergen, Norway, 2018**
