# Supplementary material for: Association between clinical frailty, illness severity and post-discharge survival: a prospective cohort study of older medical inpatients in Norway
Source: Eur Geriatr Med. 2021 Aug 21;13(2):453–61. doi: 10.1007/s41999-021-00555-8 (PMC8379589; doi:10.1007/s41999-021-00555-8)
Supplement: Supplementary file 2 — Supplementary file2 (PDF 203 KB) [file 41999_2021_555_MOESM2_ESM.pdf]

Supplemental table 1

Blood tests with reference values used to construct the FI-lab variable

	Low cut-off	High cut-off
Hemoglobin (g/dL)	Females: 11.7	15.3
	Males: 13.4	17.0
Mean corpuscular volume (fL)	82	98
Thrombocytes (10^9/L)	145	390
Leukocytes (10^9/L)	3.5	10.0
Erythrocyte sedimentation rate	Females: 1	17
	Males: 1	12
Sodium (mmol/L)	137	145
Potassium (mmol/L)	3.6	4.6
Alanine transaminase (U/L)	Females: 10	45
	Males: 10	70
Total bilirubin (µmol/L)	5	25
Urea (mmol/L)	Females: 3.1	7.9
	Males: 3.5	8.1
Creatinine (µmol/L)	Females: 45	90
	Males: 60	105
Glucose (mmol/L)	4.0	6.0
C-reactive protein (mg/L)		4
Albumin (g/L)	34	50

Normal reference values from Department of Medical Biochemistry, Oslo university hospital.

Supplementary material for «Association between clinical frailty, illness severity and post-discharge survival: A prospective cohort study of older medical inpatients in Norway», published in European Geriatric Medicine.
Authors: Andreas Engvig, Torgeir Bruun Wyller, Eva Skovlund, Marc Vali Ahmed, Trygve Sundby Hall, Kenneth Rockwood, Anne Mette Njaastad, and Bjørn Erik Neerland.
Corresponding Author: Andreas Engvig, M.D., Ph.D., andreas.engvig@gmail.com
