# Supplementary material for: Association between clinical frailty, illness severity and post-discharge survival: a prospective cohort study of older medical inpatients in Norway
Source: Eur Geriatr Med. 2021 Aug 21;13(2):453–61. doi: 10.1007/s41999-021-00555-8 (PMC8379589; doi:10.1007/s41999-021-00555-8)
Supplement: Supplementary file 3 — Supplementary file3 (PDF 227 KB) [file 41999_2021_555_MOESM3_ESM.pdf]

Supplemental table 2

Patient characteristics in relation to degree of frailty.

	Fit – well (CFS 1 to 3, n=23)	Very mild - Mild frailty (CFS 4 to 5, n=88)	Moderate frailty (CFS 6, n=44)	Severe frailty (CFS 7 to 8, n=40)	p-value*
Mean age, years (SD)	83.6 (6.2)	86.0 (5.5)	86.7 (5.6)	88.0 (5.5)	.004
Female, *N* (%)	10 (44)	57 (65)	34 (77)	21 (53)	.66
Median number of medications on discharge (IQR)	5 (4)	6 (4)	7 (4)	7 (5)	.001
Mean Charlson Comorbidity Index, (SD)	1.4 (1.5)	1.8 (1.8)	1.9 (1.7)	2.5 (1.7)	.015
Delirium during hospital stay, *N* (%)	0 (0)	24 (27)	18 (41)	20 (50)	<.001
Median length of hospital stay (IQR)	4 (5)	6 (4)	8 (6)	9 (7)	<.001
**Illness severity**					
Mean NEWS2 score (SD)	3.7 (1.9)	4.6 (2.5)	5.3 (2.6)	5.7 (3.4)	.001
Mean FI-laboratory score (SD)	.34 (.15)	.36 (.16)	.37 (.14)	.41 (.15)	.052

High clinical illness severity§, *N* (%)	5 (22)	38 (43)	28 (64)	24 (60)	.001
High laboratory illness severity§§, *N* (%)	5 (22)	22 (25)	12 (27)	14 (35)	.20
Both high clinical and laboratory illness severity, *N* (%)	3 (13)	8 (9)	7 (16)	8 (20)	.15
**Discharge destination**					
Home without need for assistance, *N* (%)	18 (78)	17 (19)	1 (2)	0 (0)	<.001
Home with assistance, *N* (%)	2 (9)	46 (52)	21 (48)	7 (18)	.32
Short-term nursing home/rehabilitation, *N* (%)	0 (0)	17 (19)	21 (48)	25 (63)	<.001
Long-term care facility, *N* (%)	1 (4)	0 (0)	0 (0)	6 (15)	.003

P-values for linear regressions of age, number of drugs at discharge, age-adjusted Charlson comorbidity index, length of stay, clinical and laboratory illness severity as dependent variables and CFS-category as independent variable. For hospital length of stay, the p-value results from a non-parametric Jonckheere-Terpstra test of trend. For binary dependent variables, p-values are from Chi square-tests of trend (linear-by-linear association). §High clinical illness severity = NEWS2-score ≥ 5. §§High laboratory illness severity = FI-value ≥

.45. Abbreviations: *N* = Number of participants. IQR = Interquartile range, i.e., difference between 25th and 75th percentile, rounded to nearest integer). SD = Standard deviation. NEWS2 = New Early Warning Score 2, maximum value during hospital stay. CFS = Clinical Frailty Scale. CI = confidence interval. FI-Laboratory = Frailty index of 14 routine laboratory tests.

Authors: Andreas Engvig, Torgeir Bruun Wyller, Eva Skovlund, Marc Vali Ahmed, Trygve Sundby Hall, Kenneth Rockwood, Anne Mette Njaastad, and Bjørn Erik Neerland.
Corresponding Author: Andreas Engvig, M.D., Ph.D., andreas.engvig@gmail.com
