# Supplementary material for: Association between clinical frailty, illness severity and post-discharge survival: a prospective cohort study of older medical inpatients in Norway
Source: Eur Geriatr Med. 2021 Aug 21;13(2):453–61. doi: 10.1007/s41999-021-00555-8 (PMC8379589; doi:10.1007/s41999-021-00555-8)
Supplement: Supplementary file 4 — Supplementary file4 (PDF 88 KB) [file 41999_2021_555_MOESM4_ESM.pdf]

Online Resource 4: The figure shows Kaplan-Meier survival curves for CFS categories 1 to 8

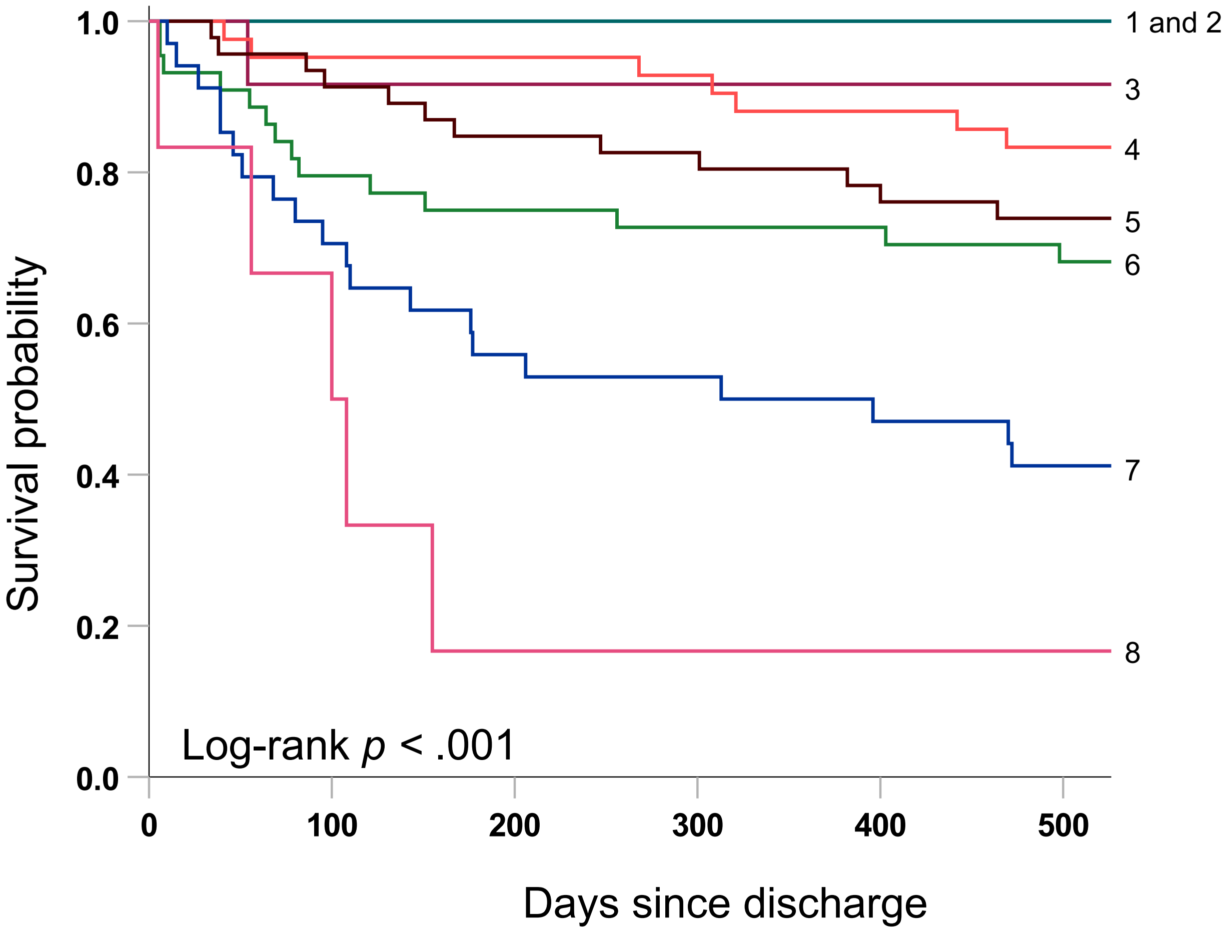

Supplementary material for «Association between clinical frailty, illness severity and post-discharge survival: A prospective cohort study of older medical inpatients in Norway», published in European Geriatric Medicine. Authors: Andreas Engvig, Torgeir Bruun Wyller, Eva Skovlund, Marc Vali Ahmed, Trygve Sundby Hall, Kenneth Rockwood, Anne Mette Njaastad, and Bjørn Erik Neerland.

Corresponding Author: Andreas Engvig, M.D., Ph.D., andreas.engvig@gmail.com
